# Long non‐coding RNA CASC7 is associated with the pathogenesis of heart failure via modulating the expression of miR‐30c

**DOI:** 10.1111/jcmm.15764

**Published:** 2020-08-29

**Authors:** Yu‐li Xu, Yang Liu, Ru‐ping Cai, Shi‐rong He, Ri‐xin Dai, Xi‐heng Yang, Bing‐hui Kong, Zhen‐bai Qin, Qiang Su

**Affiliations:** ^1^ Department of Cardiology Affiliated Hospital of Guilin Medical University Guilin China; ^2^ Department of Cardiology The Second People’s Hospital of Nanning City The Third Affiliated Hospital of Guangxi Medical University Nanning China; ^3^ Department of Cardiology The First Affiliated Hospital of Guangxi Medical University Nanning China

**Keywords:** cardiomyocyte, CASC7, heart failure, IL‐11, lncRNA, miR‐30c, miRNA

## Abstract

MiRNAs can be used as promising diagnostic biomarkers of heart failure, while lncRNAs act as competing endogenous RNAs of miRNAs. In this study, we collected peripheral blood monocytes from subjects with or without HF to explore the association between certain lncRNAs, miRNAs and HF. Heart failure patients with preserved or reduced ejection fraction were recruited for investigation. ROC analysis was carried out to evaluate the diagnostic values of certain miRNAs and lncRNAs in HF. Luciferase assays were used to study the regulatory relationship between above miRNAs and lncRNAs. LncRNA overexpression was used to explore the effect of certain miRNAs in H9C2 cells. Expression of miR‐30c was significantly decreased in the plasma and peripheral blood monocytes of patients suffering from heart failure, especially in these with reduced ejection fraction. On the contrary, the expression of lncRNA‐CASC7 was remarkably increased in the plasma and peripheral blood monocytes of patients suffering from heart failure. Both miR‐30c and lncRNA‐CASC7 expression showed a promising efficiency as diagnostic biomarkers of heart failure. Luciferase assays indicated that miR‐30c played an inhibitory role in lncRNA‐CASC7 and IL‐11 mRNA expression. Moreover, the overexpression of lncRNA‐CASC7 suppressed the expression of miR‐30c while evidently increasing the expression of IL‐11 mRNA and protein in H9C2 cells. This study clarified the relationship among miR‐30c, lncRNA‐CASC7 and IL‐11 expression and the risk of heart failure and showed that lncRNA‐CASC7 is potentially involved in the pathogenesis of HF via modulating the expression of miR‐30c.

## 1 INTRODUCTION

In general, heart failure (HF) is featured by the incapability of the heart to provide the organs and tissues in the body with an adequate amount of blood flow carrying the amount of oxygen required to complete the metabolic activities of cells.^1^ HF is usually caused by certain underlying diseases of the myocardium. Nevertheless, heart valve disorders, endocardial heart abnormalities and other pericardial diseases influencing in the rhythm and rate of heart beat can also lead to cardiac malfunctions.^1^

Long non‐coding RNA (lncRNA) belongs to a type of RNA transcripts with no coding capability. In fact, lncRNAs are pervasively distributed in the genome of humans and many other species.^2^, ^3^, ^4^ Past studies showed that lncRNAs can carry out critical functions during a wide range of fundamental and physiological processes including the regulation of DNA transcription, gene expression, cell apoptosis and modification of chromatin.^5^, ^6^ In addition, lncRNAs are involved in the onset and progression of a wide range of human disorders.^7^, ^8^, ^9^

Cancer Susceptibility Candidate 7 (CASC7) is an lncRNA of about 9 kb in length.^10^ As an lncRNA frequently seen in nuclear fractions, CASC7 can bind to large‐sized polysomal complexes located in the cytoplasm to influence the regulatory role of CASC7 in gene translation.^10^ In SY‐SH‐5Y cells, it was shown that NaSH can regulate the expression of miR‐30c in the presence of CASC7, subsequently preventing the apoptosis of SY‐SH‐5Y cells induced by the treatment with OGD/R.^11^

In a study carried out by Panizo et al,^12^ the expression of miR‐30c was a potential serum biomarker for cardiac fibrosis, which could be regulated by vitamin D receptor activators and thus attenuate cardiac fibrosis. In addition, patients suffering from polycythemia vera show abnormal levels of miR‐30c expression in their reticulocytes, while the down‐regulated expression of miR‐30c was observed in a wide range of human malignancies, including gastric cancer, breast cancer, colorectal cancer and bladder cancer.^13^, ^14^, ^15^, ^16^ In addition, miR‐30c can affect the expression levels of PAI‐1 in human endothelial cells by directly binding to the 3′ untranslated region (UTR) of PAI‐1.^17^

In a study carried out by Lai et al, miR‐30e was reported to play a cardio‐protective role in HF via the miR‐30e/Beclin‐1 signalling axis, thus preventing the apoptosis of cardiomyocytes induced by autophagy, and the inhibition of apoptosis and autophagy of cardiomyocytes by miR‐30e contributes to the increased cardiac functions in cardiotoxicity mediated by doxorubicin and ACE2.^18^

It has been found that three lncRNAs (lncRNA‐CCAT1, lncRNA‐CASC7, lncRNA‐ AK017368) are competing endogenous RNAs of miR‐30c, which can be used as a diagnostic biomarker of HF.^11^, ^19^, ^20^ Moreover, the expression if IL‐11 was also reported to down‐regulate the expression of miR‐30c in primary breast tumours while the reduction of IL‐11 was also associated with the relapse‐free survival in breast cancer patients.^21^ In this study, we collected peripheral blood from subjects with or without HF and preserved EF to investigate the role of several lncRNAs and miRNAs in HF.

## 2 MATERIALS AND METHODS

### 2.1 Human subjects and sample collection

A total of 186 participants were recruited in our study. These 186 patients consisted of 62 subjects without heart failure (control group), 62 participants diagnosed with heart failure and preserved ejection fraction (HF + pEF group) and 62 participants diagnosed with heart failure and reduced ejection fraction (HF + rEF group) and all those participants were enrolled in Affiliated Hospital of Guilin Medical University during February 2015 to March 2018. 5 mL peripheral blood samples were collected from each subject. Then, the demographic and clinicopathological characteristics of the participants, such as their age, gender, systolic blood pressure (SBP), diastolic blood pressure (DBP), heart rate, body mass index, hypertension, diabetes mellitus, dyslipidaemia, ischaemic heart disease, atrial fibrillation, brain natriuretic peptide (BNP), echocardiography, ejection fraction, interventricular septum, posterior wall, left ventricular mass index (LVMI), left atrial volume index (LAVI), deceleration time, e’ (lat), peak E and the ratio of transmitral early peak velocity to E' (E/e’), were collected and compared among the three groups. This study was approved by the Ethics Committee of Affiliated Hospital of Guilin Medical University and informed consent has been obtained upon the initiation of this study.

### 2.2 RNA isolation and real‐time PCR

The collected peripheral blood samples were treated using a commercial kit to separate plasma and isolate peripheral blood monocytes. Then, the expression of several miRNAs, that is miR‐30c, miR‐146a, miR‐221, miR‐328 and miR‐375, as well as the expression of several lncRNAs, that is lncRNA‐CCAT1, lncRNA‐CASC7 and lncRNA‐AK017368, in the plasma and peripheral blood monocytes was measured using real‐time PCR. In brief, all samples were treated with a mirVana kit (Ambion, Applied Biosystems) to isolate total RNA, which was then mixed with reagents in a kit of Universal complementary DNA synthesis (Thermo Fisher Scientific), a SYBR Green master mix (Applied Biosystems) and a primer sets specifically designed for PCR. The real‐time PCR was performed using 96‐well plates on 7900HT real‐time PCR instrument (Invitrogen). The final calculation of relative expression of miR‐30c (Forward: 5′‐ACATCCTACACTCTCAGC‐3′; Reverse: 5′‐ GAACATGTCTGCGTATCTC‐3′), miR‐146a (Forward: 5′‐GAGAACTGAATTCCATGG‐3′; Reverse: 5′‐GAACATGTCTGCGTATCTC‐3′), miR‐221 (Forward: 5′‐ACCTGGCATACAATGTAG‐3′; Reverse: 5′‐GAACATGTCTGCGTATCTC‐3′), miR‐328 (Forward: 5′‐CCTCTCTGCCCTTCCG‐3′; Reverse: 5′‐GAACATGTCTGCGTATCTC‐3′), miR‐375 (Forward: 5′‐TTTGTTCGTTCGGCTCG‐3′; Reverse: 5′‐GAACATGTCTGCGTATCTC‐3′), lncRNA‐CCAT1 (Forward: 5′‐GCCGTGTTAAGCATTGCGAA‐3′; Reverse: 5′‐AGAGTAGTGCCTGGCCTAGA‐3′), lncRNA‐CASC7 (Forward: 5′‐TGTTTGATTCCTCGTCGCT‐3′; Reverse: 5′‐GGCACCGTAATGGCACTG‐3′), lncRNA‐ AK017368 (Forward: 5′‐TGGACGTAAGGGAGTGCAGA‐3′; Reverse: 5′‐TAGGACTCACCTGCCTTGGA‐3′) and IL‐11 mRNA (Forward: 5′‐ GGACCACAACCTGGATTCCCTG‐3′; Reverse: 5′‐ AGTAGGTCCGCTCGCAGCCTT‐3′) were done using the ΔΔCt method, and all results were normalized to the expression of endogenous control U6 (Forward: 5′‐CTCGCTTCGGCAGCACAT‐3′; Reverse: 5′‐TTTGCGTGTCATCCTTGCG‐3′) and GAPDH (Forward: 5′‐ GTCTCCTCTGACTTCAACAGCG‐3′; Reverse: 5′‐ ACCACCCTGTTGCTGTAGCCAA‐3′). We have performed the validation test for SYBR green‐based qPCR following the method described previously.^22^

### 2.3 Cell culture and transfection

H9C2 cells, a cell line of embryonic rat cardiomyocytes derived from embryonic BD1X rat heart tissues, were acquired from ATCC and maintained in a modified DMEM media (Gibco, Thermo Fisher Scientific) supplemented with appropriate antibiotics and 10% foetal bovine serum. The cultured cells were incubated at 37°C in 5% CO_2_ and saturated humidity. When the cultured H9C2 cells entered the phase of logarithmic growth, they were trypsinized and made into a single cell suspension. Then, for each one of three lncRNAs to be tested, that is lncRNA‐CASC7, lncRNA‐CCAT1 and lncRNA‐ AK017368, the H9C2 cells were divided into two groups, that is 1. Negative control (NC) and 2. p‐lncRNA‐CASC7; 1. NC and 2. p‐lncRNA‐CCAT1; and 1.NC and 2. p‐lncRNA‐ AK017368. In the next step, the cells in various groups were transfected with respective pcDNA 3.1 plasmids of lncRNAs at 30nM using Lipofectamine 3000 (Invitrogen) in accordance with the standard transfection protocol provided by the manufacturer. All transfected cells were harvested at 48 hours after transfection to analyse the expression of target genes.

### 2.4 Vector construction, mutagenesis and luciferase assay

Target Scan software was utilized in this study to search for potential miR‐30c binding sites located in lncRNA‐CCAT1, lncRNA‐CASC7 and lncRNA‐AK017368. Then, based on the sequences obtained from the Target Scan analysis, the sequences of above 3 lncRNAs containing the miR‐30c binding sites were cloned into pcDNA luciferase vectors to obtain wild‐type lncRNA‐CCAT1, lncRNA‐CASC7 and lncRNA‐AK017368 vectors. At the same time, using a commercial site‐directed mutagenesis assay kit (Quick Change II, Stratagene), site‐directed mutagenesis was carried out to generate a single mutation in the miR‐30c binding sites located in lncRNA‐CCAT1, lncRNA‐CASC7 and lncRNA‐AK017368, respectively. In the next step, H9C2 cells were co‐transfected with miR‐30c mimics and wild‐type or mutant vectors of lncRNA‐CCAT1, lncRNA‐CASC7 or lncRNA‐AK017368. At 48 hours after transfection, the luciferase activity of transfected cells in each group was assayed using a Bright Glo luciferase assay system (Promega) in accordance with the standard protocol provided by the manufacturer.

### 2.5 Western blot analysis

Cell samples were collected and rinsed two times using cold PBS (Gibco, Thermo Fisher Scientific), which were subsequently lysed with a protease inhibitor RIPA buffer (Gibco, Thermo Fisher Scientific). The isolated protein samples were then separated using 10% SDS‐PAGE and blotted onto PVDF membranes (Thermo Fisher Scientific). After being incubated with anti‐IL‐11 (dilution: 1:2000, ab187167, Abcam) primary antibodies and HRP secondary antibodies (dilution: 1:5000, ab6721, Abcam) following a conventional protocol, the PVDF membranes were developed using an ECL kit (Thermo Fisher Scientific) and β‐actin was used as the internal reference for the densitometric analysis.

### 2.6 Statistical analysis

One‐way analysis of variance (ANOVA) was used to compare statistical differences among various groups. The receiver operating characteristic (ROC) analysis was performed to determine the diagnostic values of various miRNA and lncRNA candidates. The effects of potential confounding factors such as age, gender, NYHA class, BNP was evaluated using multiple variate analysis. The statistical power of analysis is 0.97 (http://www.biosoft.hacettepe.edu.tr/easyROC/). All statistical analyses were carried out using SPSS version 21.0 statistical software. All statistical differences were compared using a level of significance of 0.05.

## 3 RESULTS

### 3.1 Demographic and clinicopathological parameters of the participants recruited in this study

The demographic and clinicopathological characteristics of the 186 participants were collected and summarized in Table 1. Accordingly, no significant difference was observed with respect to gender, heart rate, dyslipidaemia and interventricular septum. However, the remaining characteristics showed obvious differences among the three groups.

**TABLE 1:** Demographic and clinicopathological characteristics of the participants of this study

Parameter	Healthy control (N = 62)	HF + pEF (N = 62)	HF + rEF (N = 62)	*P* value
Age (y)	70.8 ± 6.5	73.5 ± 6.6	68.7 ± 7.4	.561
Sex, Male (%)	36 (58.1)	36 (58.1)	34 (54.8)	.817
SBP (mm Hg)	137.3 ± 5.7	124.4 ± 6.2	116.8 ± 5.6	<.01
DBP (mm Hg)	84.2 ± 5.2	74.2 ± 6.5	68.5 ± 5.9	<.01
Heart rate, b.p.m.	73.2 ± 6.4	78.2 ± 6.1	73.2 ± 5.4	.569
BMI (kg/m^2^)	28.6 ± 2.6	32.8 ± 2.1	28.9 ± 3.1	<.01
Hypertension, %	48 (77.4)	51 (82.3)	23 (37.4)	.450
Diabetes mellitus, %	13 (21.0)	18 (29.0)	8 (12.9	<.01
Dyslipidaemia, %	37 (59.7)	35 (56.5)	40 (64.5)	.224
Ischaemic heart disease, %	16 (25.8)	30 (48.4)	41 (66.1)	<.01
Atrial fibrillation, %	7 (11.3)	48 (77.4)	35 (56.5)	<.01
BNP, pg/mL	32.5 ± 4.5	235.0 ± 44.1	126.7 ± 33.7	<.01
Echocardiography				
Ejection fraction	66.0 ± 10.2	64.8 ± 13.6	37.6 ± 7.9	<.01
Interventricular septum, mm	11.8 ± 1.6	12.6 ± 1.1	11.0 ± 0.7	.070
Posterior wall, mm	9.7 ± 3.5	10.9 ± 3.3	9.8 ± 2.2	<.01
LVMI, g/m^2^	96.2 ± 16.7	118.1 ± 13.0	127.4 ± 17.7	<.01
LAVI, mL/m^2^	30.8 ± 9.4	51.1 ± 3.5	46.0 ± 7.1	<.01
Deceleration time, ms	197.8 ± 36.6	218.0 ± 27.7	235.7 ± 40.1	<.01
e'(lat), cm/s	8.5 ± 2.4	9.6 ± 1.8	8.1 ± 2.0	<.05
Peak E, cm/s	74.8 ± 28.8	96.2 ± 22.3	66.5 ± 29.3	<.01
E/e' ratio	9.5 ± 1.6	11.8 ± 2.9	9.6 ± 1.9	<.01

Abbreviations: BMI, body mass index; BNP, brain natriuretic peptide; DBP, diastolic blood pressure; E/e’ ratio, transmitral early peak velocity to E' ratio; LAVI, left atrial volume index; LVMI, left ventricular mass index; SBP, systolic blood pressure.

### 3.2 MiR‐30c was down‐regulated in the plasma and peripheral blood monocytes of heart failure patients

**FIGURE 1:**
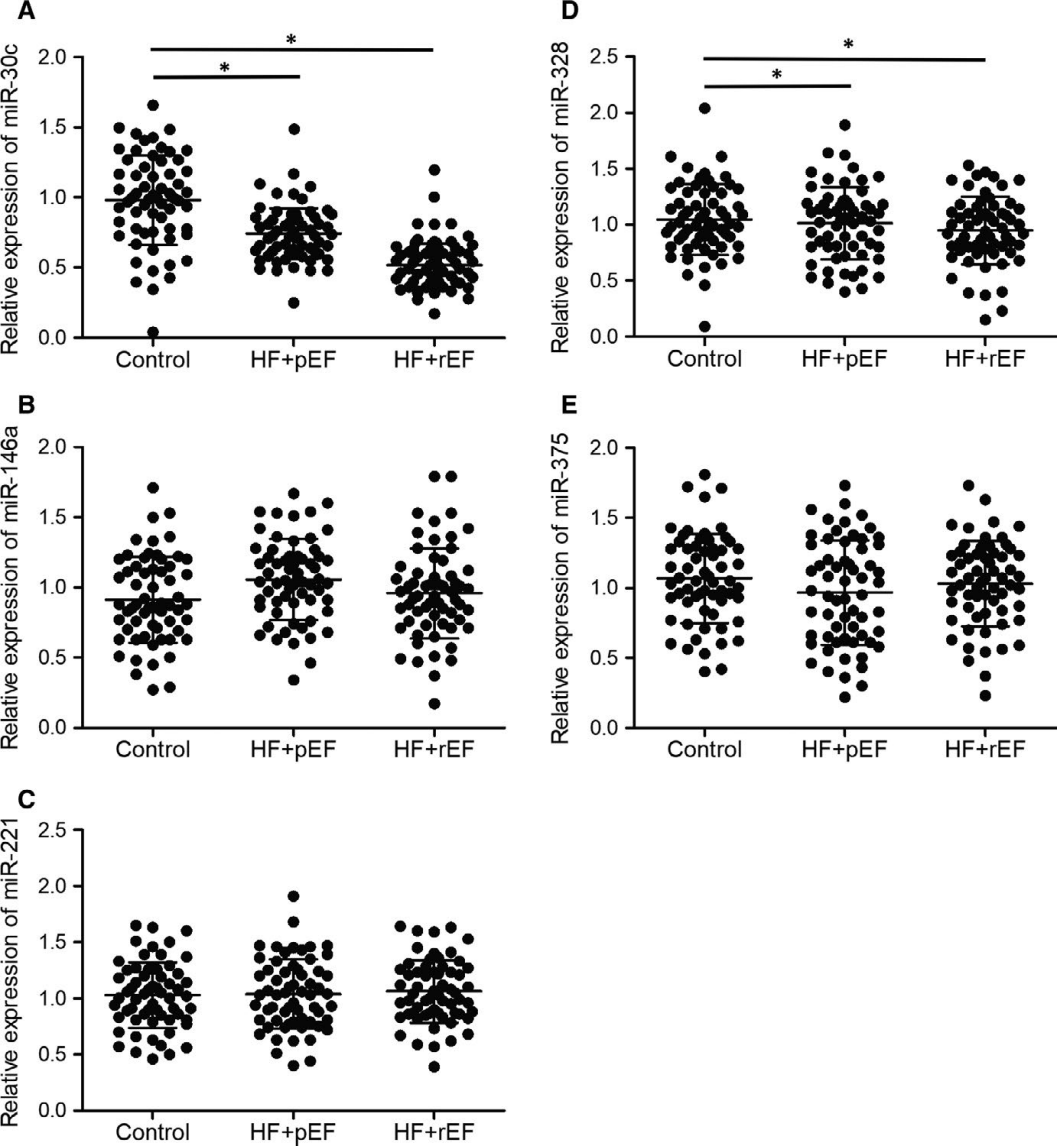
miR‐30c was down‐regulated in the plasma of heart failure patients (*P value <0.05 vs. control group; HF: heart failure; pEF: preserved ejection fraction; rEF: reduced ejection fraction). A, Expression of miR‐30c was decreased in the plasma of HFpEF and HFrEF patients. B, Expression of miR‐146a was not significantly changed in the plasma of HFpEF, HFrEF and control groups. C, Expression of miR‐221 was not significantly changed in the plasma of HFpEF, HFrEF and control groups. D, Expression of miR‐328 was not significantly changed in the plasma of HFpEF, HFrEF and control groups. E, Expression of miR‐375 was not significantly changed in the plasma of HFpEF, HFrEF and control groups

**FIGURE 2:**
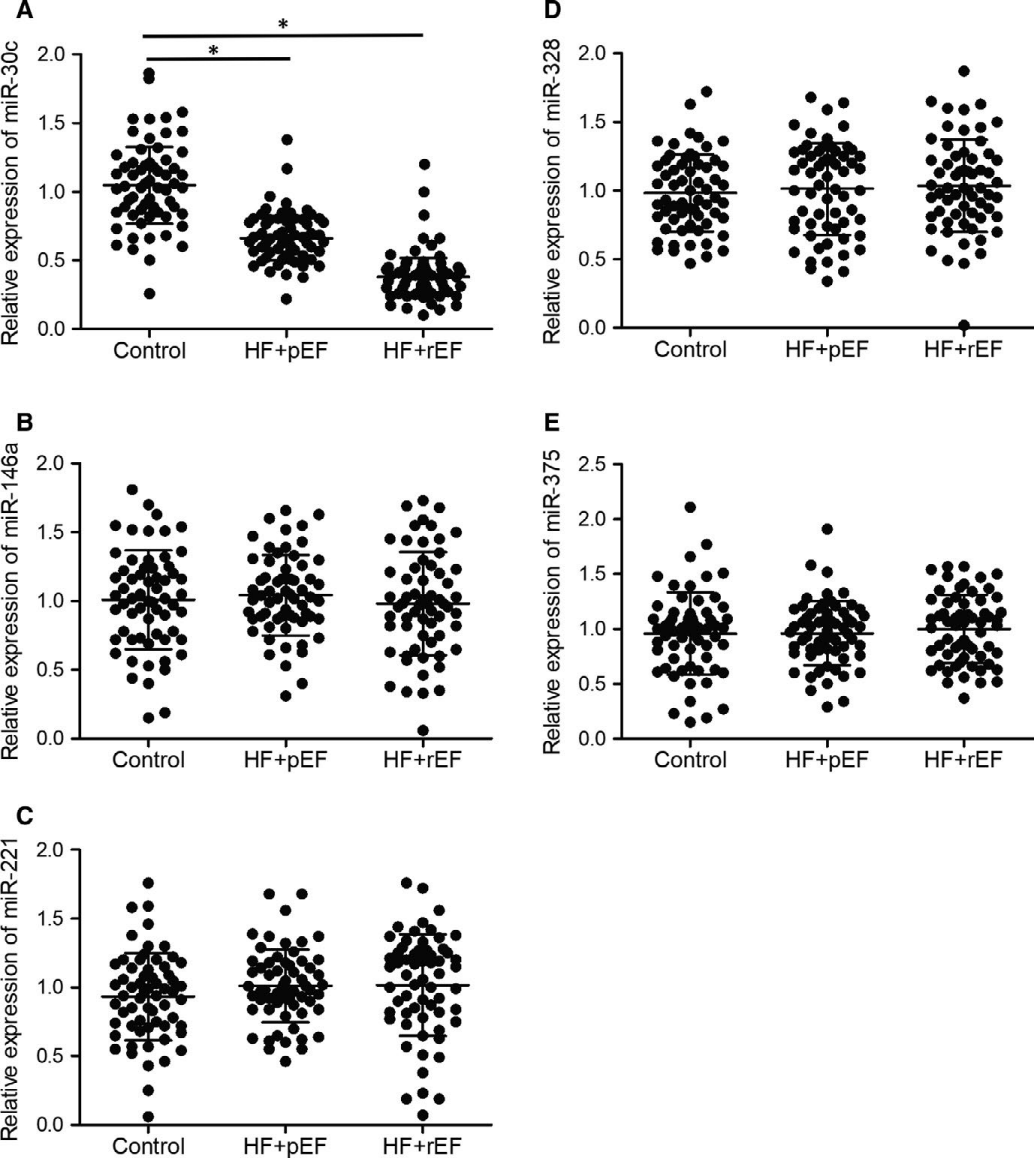
miR‐30c was down‐regulated in the peripheral blood monocytes of heart failure patients (*P value <0.05 vs. control group; HF: heart failure; pEF: preserved ejection fraction; rEF: reduced ejection fraction). A, Expression of miR‐30c was decreased in the peripheral blood monocytes of HFpEF and HFrEF patients. B, Expression of miR‐146a was not significantly different in the peripheral blood monocytes of HFpEF, HFrEF and control groups. C, Expression of miR‐221 was not significantly changed in the peripheral blood monocytes of HFpEF, HFrEF and control groups. D, Expression of miR‐328 was not significantly changed in the peripheral blood monocytes of HFpEF, HFrEF and control groups. E, Expression of miR‐375 was not significantly changed in the peripheral blood monocytes of HFpEF, HFrEF and control groups

MiR‐30c expression was significantly decreased in HFpEF and HFrEF groups (Figures 1A and 2A). However, there was no remarkable difference in the expression of miR‐146a (Figures 1B and 2B), miR‐221 (Figures 1C and 2C), miR‐328 (Figures 1D and 2D) and miR‐375 (Figures 1F and 2F) in plasma and peripheral blood monocytes of the control, HFpEF and HFrEF patients. These results indicated that miR‐30c is a potential biomarker for HF diagnosis and can differentiate HFpEF from HFrEF.

### 3.3 MiR‐30c was a promising biomarker for heart failure diagnosis

**FIGURE 3:**
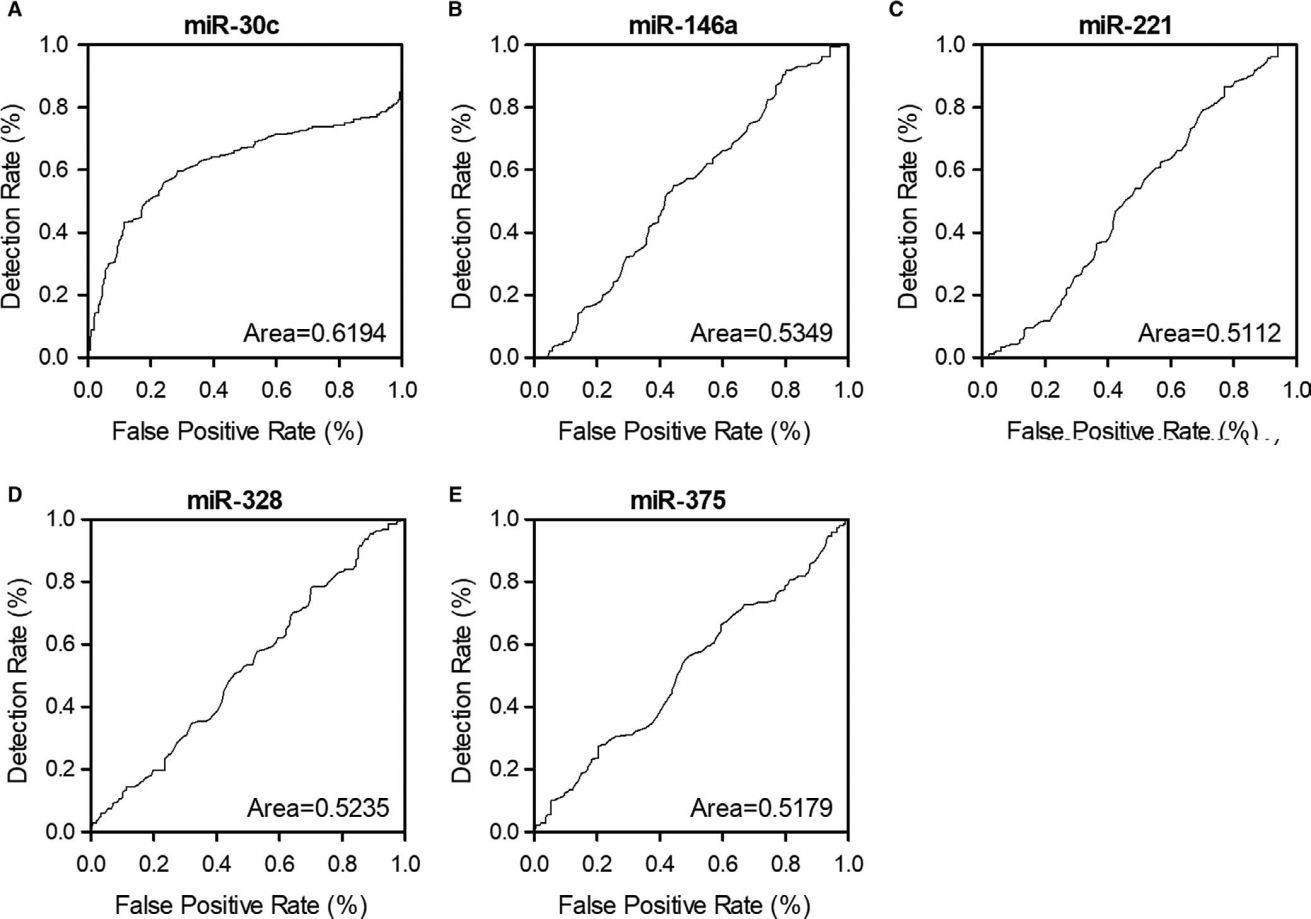
ROC analysis for the diagnostic value of multiple candidate miRNAs indicated that miR‐30c was a promising biomarker for heart failure diagnosis. A, ROC analysis for the diagnostic value of miR‐30c in heart failure. B, ROC analysis showed that miR‐146a was unable to differentiate heart failure from normal control. C, ROC analysis showed that miR‐221 was unable to differentiate heart failure from normal control. D, ROC analysis showed that miR‐328 was unable to differentiate heart failure from normal control. E, ROC analysis showed that miR‐375 was unable to differentiate heart failure from normal control

To further explore the diagnostic value of miRNAs in heart failure, a ROC analysis was performed for the above five miRNA candidates. As shown in Figure 3, the AUC of miR‐30c was 0.62 (Figure 3A), while the AUC values were about 0.5 for miR‐146a (Figure 3B), miR‐221 (Figure 3C), miR‐328 (Figure 3D) and miR‐375 (Figure 3F), indicating that miR‐30c can be used to diagnose HF while miR‐146a, miR‐221, miR‐328 and miR‐375 are unable to differentiate HF from control.

### 3.4 LncRNA‐CASC7 was up‐regulated in the plasma and peripheral blood monocytes of heart failure patients

**FIGURE 4:**
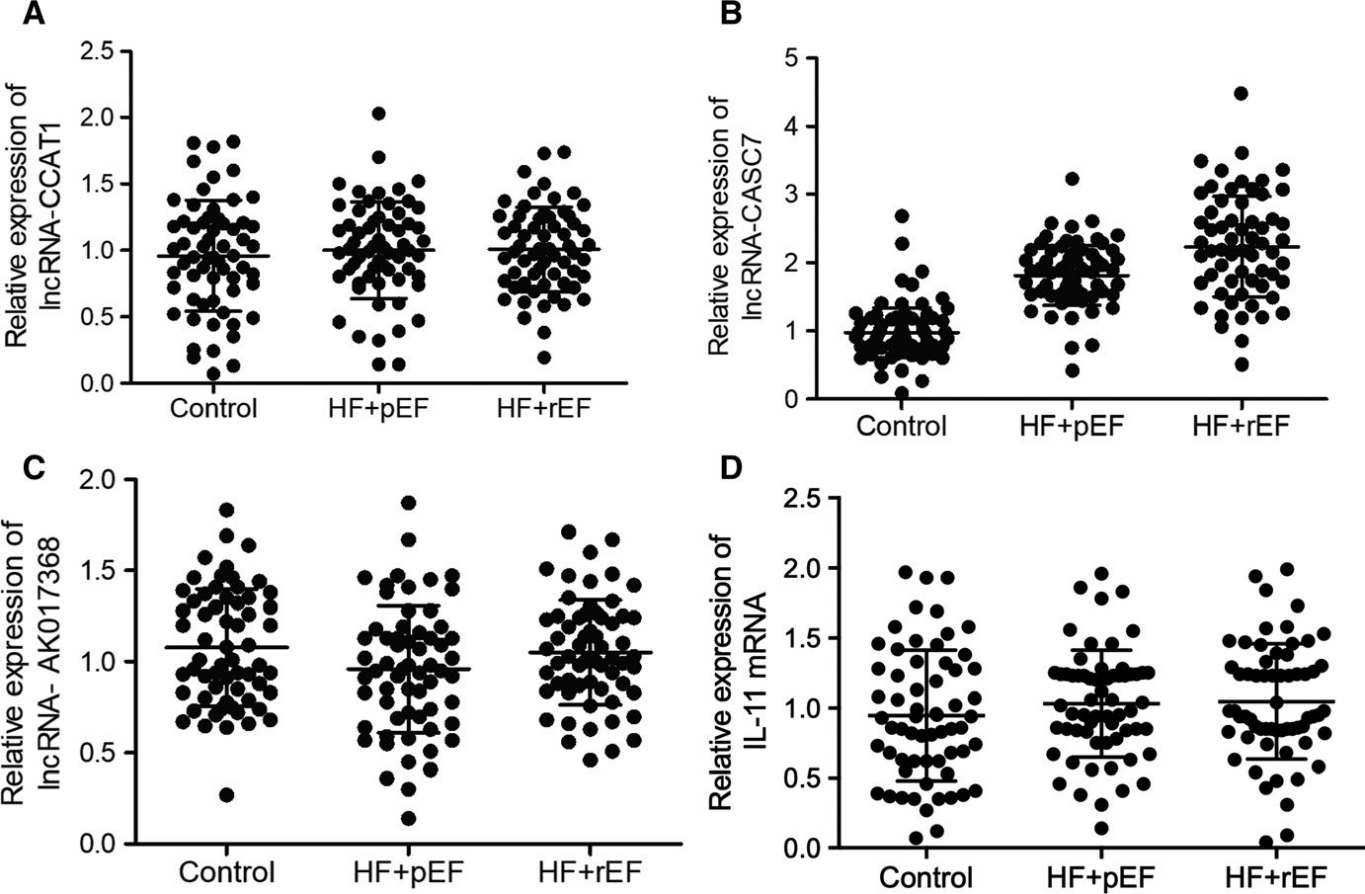
lncRNA‐CASC7 was up‐regulated in the plasma of heart failure patients (*P value <0.05 vs. control group; HF: heart failure; pEF: preserved ejection fraction; rEF: reduced ejection fraction). A, Expression of lncRNA‐CCAT1 was not significantly different in the plasma of HFpEF, HFrEF and control groups. B, Expression of lncRNA‐CASC7 was elevated in the plasma of HFpEF and HFrEF patients. C, Expression of lncRNA‐AK017368 was not significantly different in the plasma of HFpEF, HFrEF and control groups. D, Expression of IL‐11 mRNA was not significantly different in the plasma of HFpEF, HFrEF and control groups

**FIGURE 5:**
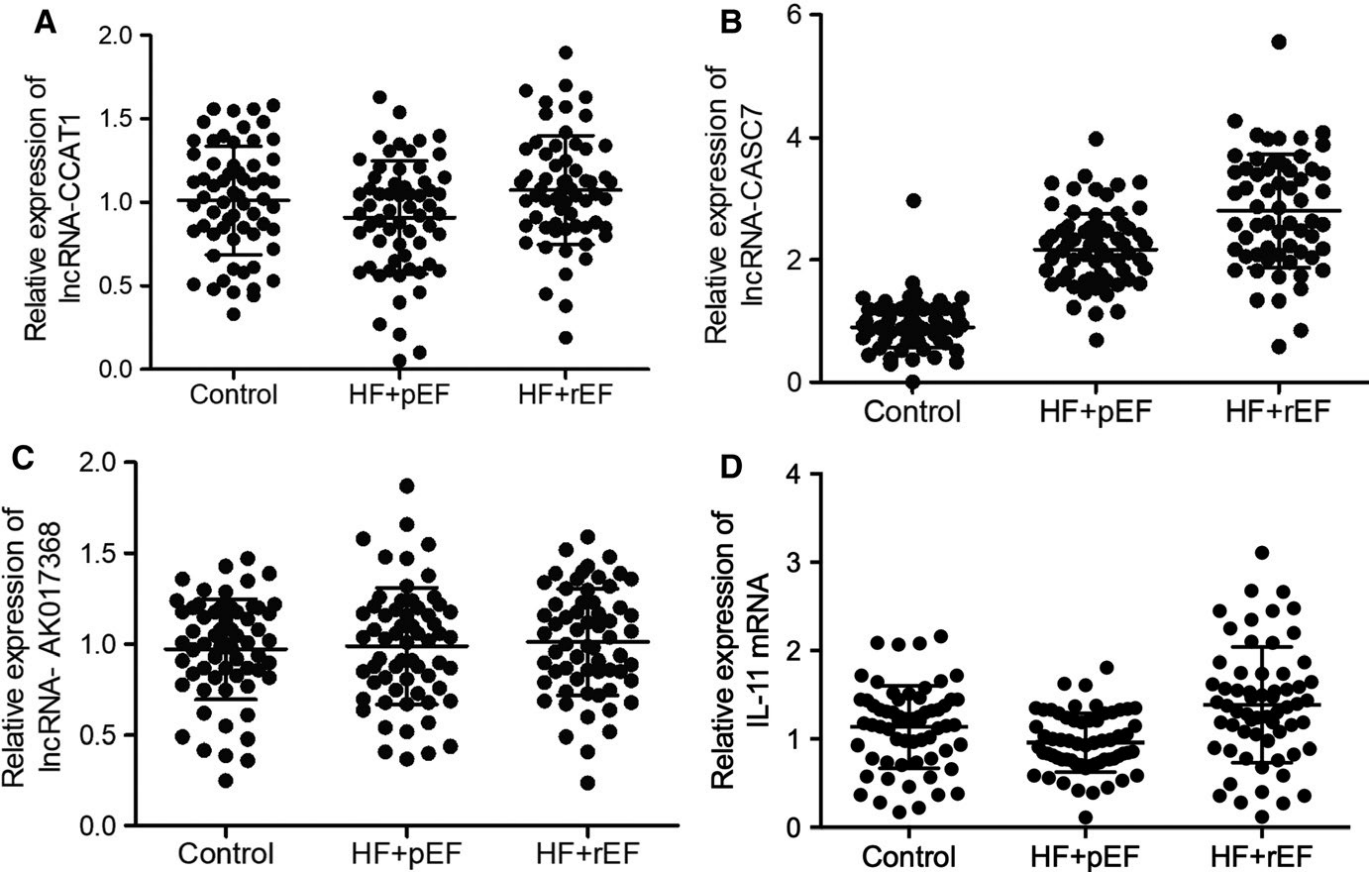
lncRNA‐CASC7 was up‐regulated in the peripheral blood monocytes of heart failure patients (*P value <0.05 vs. control group; HF: heart failure; pEF: preserved ejection fraction; rEF: reduced ejection fraction). A, Expression of lncRNA‐CCAT1 was not significantly different in the peripheral blood monocytes of HFpEF, HFrEF and control groups. B, Expression of lncRNA‐CASC7 was elevated in the peripheral blood monocytes of HFpEF and HFrEF patients. C, Expression of lncRNA‐AK017368 was not significantly different in the peripheral blood monocytes of HFpEF, HFrEF and control groups. D, Expression of IL‐11 mRNA was not significantly different in the peripheral blood monocytes of HFpEF, HFrEF and control groups

It was reported that lncRNAs play crucial roles in miRNA regulation. In this study, the expression of three candidate lncRNAs was evaluated using plasma and peripheral blood monocyte samples collected from the patients enrolled in this study. We found that the expression of lncRNA‐CASC7 was notably elevated in both plasma (Figure 4B) and peripheral blood monocytes (Figure 5B) of HFpEF and HFrEF groups. However, the expression of lncRNA‐CCAT1 and lnc‐AK017368 remained unchanged in the plasma (Figures 4A and 5A) and PBMCs (Figures 4C and 5C) of patients in HFpEF and HFrEF groups. Additionally, the expression of IL‐11 mRNA in the plasma (Figure 4D) and PBMCs (Figure 5D) was similarly unchanged among different groups as well.

### 3.5 LncRNA‐CASC7 was negatively correlated with miR‐30

**FIGURE 6:**
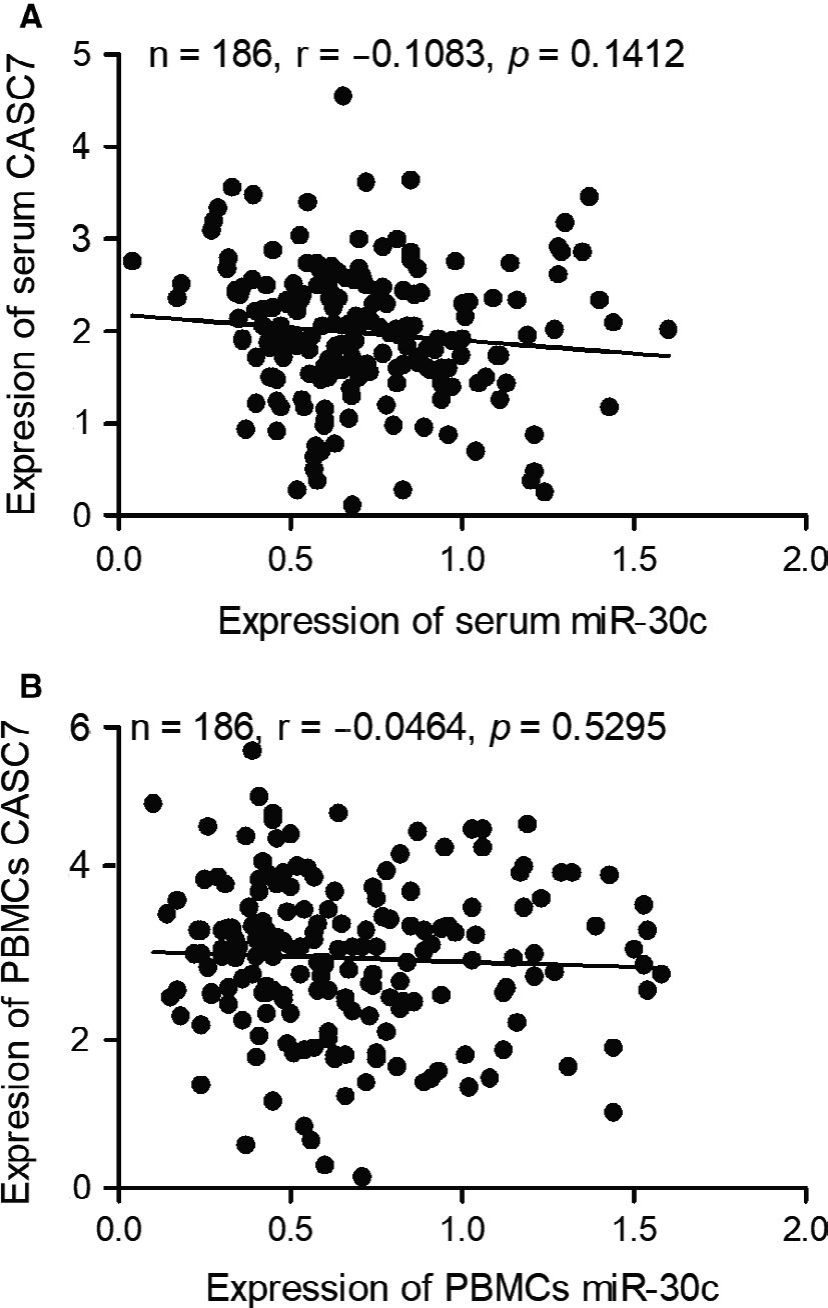
Pearson's correlation analysis indicated a negative relationship between CASC7 and miR‐30. A, Pearson's correlation analysis of serum CASC7 and serum miR‐30 showed a negative relationship between CASC7 and miR‐30. B, Pearson's correlation analysis of cellular CASC7 and cellular miR‐30 in PBMCs showed a negative relationship between CASC7 and miR‐30

Moreover, Pearson's correlation analysis was used to study the correlation between lncRNA‐CASC7 and miR‐30 in serum samples (Figure 6A) and peripheral blood monocytes (Figure 6B). Accordingly, it was demonstrated that expression of lncRNA‐CASC7 and expression of miR‐30 was negatively correlated, although no statistical significant was demonstrated.

### 3.6 LncRNA‐CASC7 was a biomarker for heart failure diagnosis

**FIGURE 7:**
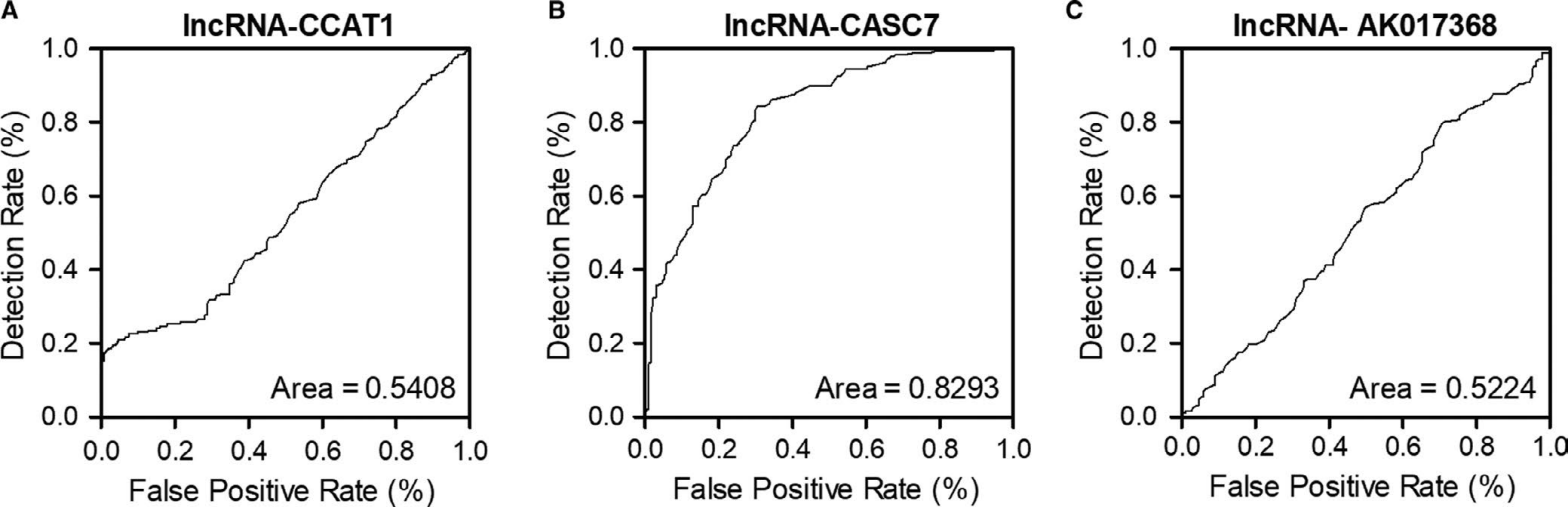
ROC analysis for the diagnostic value of multiple candidate lncRNAs indicated that lncRNA‐CASC7 was a promising biomarker for heart failure diagnosis. A, ROC analysis showed that lncRNA‐CCAT1 was unable to differentiate heart failure from normal control. B, ROC analysis of the diagnostic value of lncRNA‐CASC7 in heart failure. C, ROC analysis showed that lncRNA‐AK017368 was unable to differentiate heart failure from normal control

A ROC analysis was used to evaluate the values of lncRNA‐CCAT1, lncRNA‐CASC7 and lncRNA‐AK017368 in HF diagnosis. The AUCs for lncRNA‐CCAT1 (Figure 7A) and lncRNA‐AK017368 (Figure 7C) were 0.54 and 0.55, while the AUC for lncRNA‐CASC7 (Figure 7B) was 0.85. These results demonstrated that lncRNA‐CASC7 was capable of differentiating HF from the controls.

### 3.7 MiR‐30c inhibited lncRNA‐CASC7 and IL‐11 expression

**FIGURE 8:**
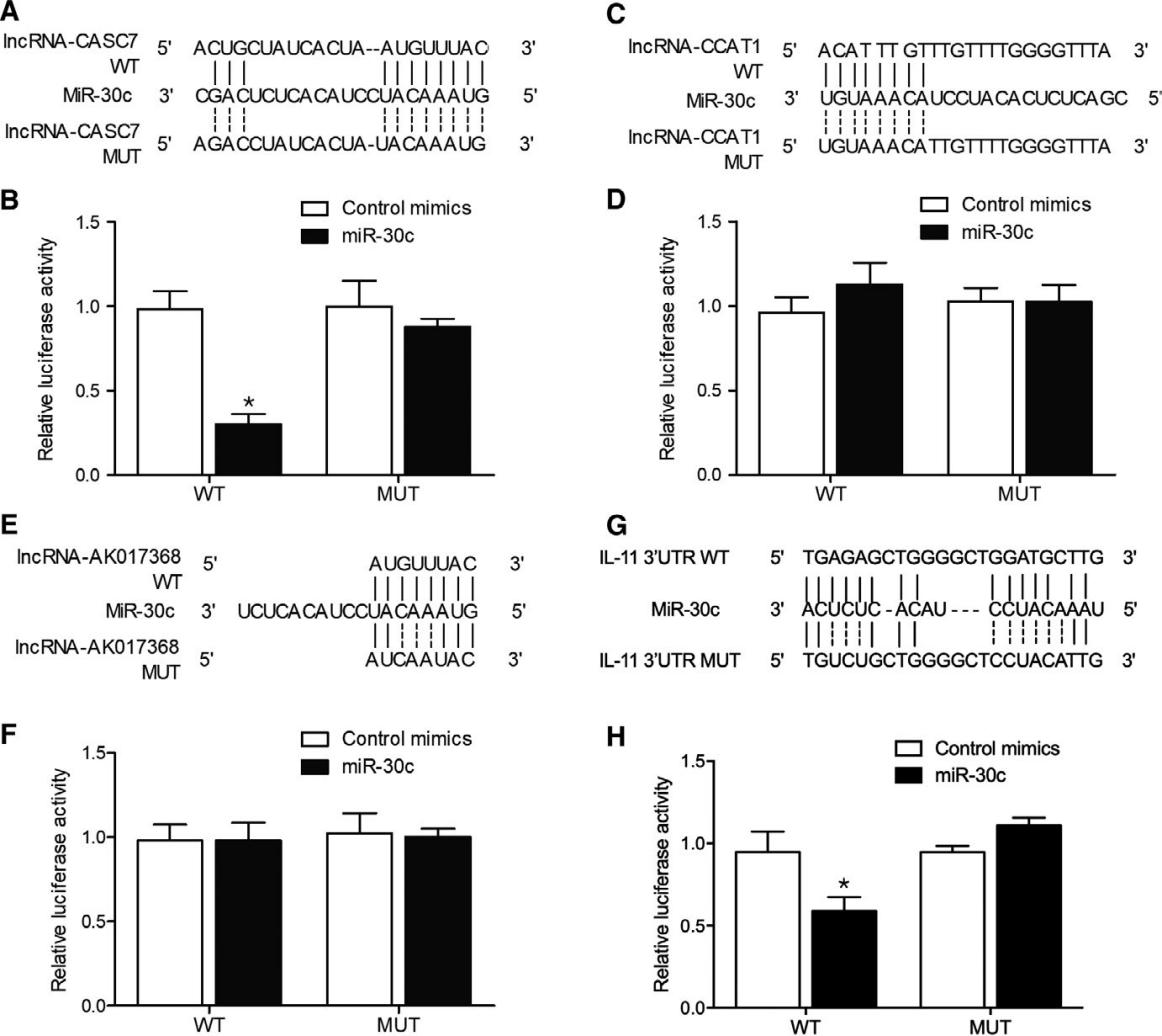
miR‐30c inhibited the luciferase activity of the lncRNA‐CCAT1 and IL‐11 3′UTR plasmid (*P value <0.05 vs. control mimics group; WT: wild type; MUT: mutant). A, Sequence analysis showed a target of miR‐30c in lncRNA‐CASC7 (Binding site: 235bp‐257bp). B, Luciferase activity of WT lncRNA‐CASC7 vector was inhibited by miR‐30c mimics in H9C2 cells. C, Sequence analysis showed a target of miR‐30c in lncRNA‐ CCAT1 (Binding site: 121bp‐145bp). D, Luciferase activity of WT and MT lncRNA‐CASC7 vectors was not affected by miR‐30c mimics in H9C2 cells. E, Sequence analysis showed a target of miR‐30c in lncRNA‐ AK017368 (Binding site: 235bp‐257bp). F, Luciferase activity of WT lncRNA‐CASC7 vector was not suppressed by miR‐30c mimics in H9C2 cells. G, Sequence analysis showed a target of miR‐30c in IL‐11 3′UTR (Binding site: 367bp‐390bp). H, Luciferase activity of WT IL‐11 3′UTR vector was obstructed by miR‐30c mimics in H9C2 cells

It has been reported that lncRNA‐CCAT1, lncRNA‐CASC7 and lncRNA‐AK017368 were competing endogenous lncRNAs of miR‐30c. Our sequence analysis also confirmed the presence of potential targeting sites of miR‐30c on lncRNA‐CCAT1, lncRNA‐CASC7 and lncRNA‐AK017368 (Figure 8A, 8C, 8E). Luciferase plasmids containing wild‐type (WT) and mutant (MT) lncRNAs were established and co‐transfected with miR‐30c mimics into H9C2 cells. The results clearly showed that miR‐30c had a remarkable inhibitory effect on the expression of WT lncRNA‐CASC7, but showed no suppressive effect on the expression of MT lncRNA‐CASC7 (Figure 8B). However, no significant difference was observed in H9C2 cells transfected with lncRNA‐CCAT1 (Figure 8D) and lncRNA‐AK017368 (Figure 8F). Similarly, the luciferase assay was repeated to observe the relationship between miR‐30c and IL‐11 mRNA since potential targeting sites of miR‐30c were detected on 3′UTR of IL‐11 mRNA (Figure 8G). Accordingly, the results showed that luciferase activity was only inhibited upon the co‐transfection of wild‐type IL‐11 3′UTR and miR‐30c mimics (Figure 8H), indicating the expression of IL‐11 mRNA was targeted by miR‐30c in H9C2 cells.

### 3.8 LncRNA‐CASC7 suppressed the expression of miR‐30c in H9C2 cells

**FIGURE 9:**
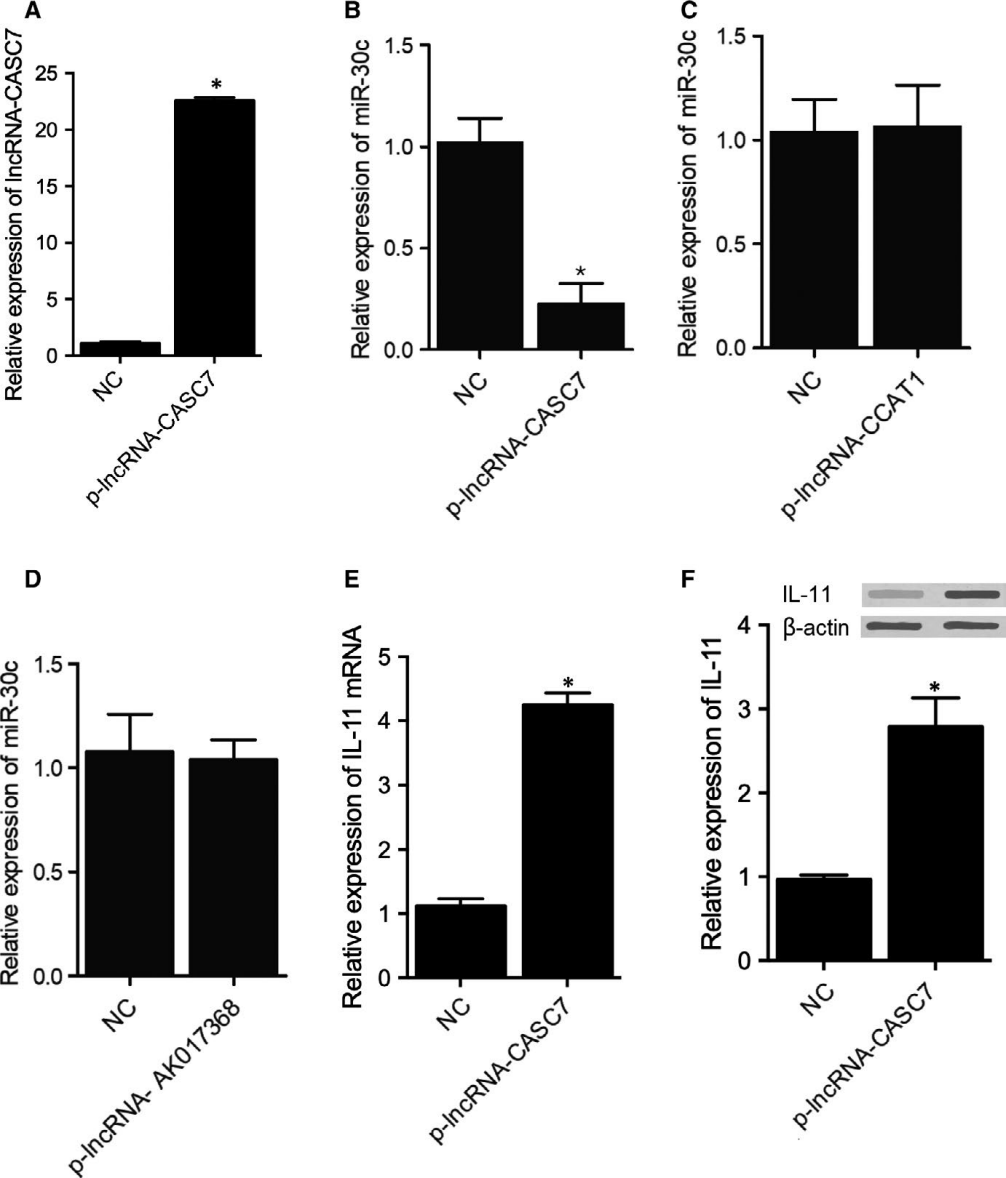
lncRNA‐CASC7 suppressed the expression of miR‐30c in H9C2 cells (*P value <0.05 vs. NC group; NC: negative control). A, The successful transfection of plasmids carrying lncRNA‐CASC7 led to the overexpression of lncRNA‐CASC7 in H9C2 cells. B, Overexpression of lncRNA‐CASC7 reduced the expression of miR‐30c in H9C2 cells. C, Expression of miR‐30c was decreased in H9C2 cells transfected with lncRNA‐ CCAT1. D, Expression of miR‐30c was not affected in H9C2 cells transfected with lncRNA‐ AK017368. E, Overexpression of lncRNA‐CASC7 increased the expression of IL‐11 mRNA in H9C2 cells. F, Overexpression of lncRNA‐CASC7 increased the expression of IL‐11 in H9C2 cells

To further investigate the regulatory relationship between miR‐30c and above lncRNAs, lncRNA‐CASC7 (Figure 9A), lncRNA‐CCAT1 (data not shown) and lncRNA‐AK017368 (data not shown) were transfected into H9C2 cells before the expression of miR‐30c was examined. Accordingly, the overexpression of lncRNA‐CASC7 evidently repressed the expression of miR‐30c in H9C2 cells (Figure 9B). On the contrary, transfection of lncRNA‐CCAT1 (Figure 9C) and lncRNA‐AK017368 (Figure 9D) showed no apparent effect on the expression of miR‐30c in H9C2 cells. Also, the expression of IL‐11 mRNA (Figure 9E) and protein (Figure 9F) was both significantly elevated by the overexpression of lncRNA‐CASC7.

## 4 DISCUSSION

Many lncRNAs can function as competing endogenous RNAs (ceRNAs) to modulate the intracellular activity of their target miRNAs.^23^ For example, CRNDE is an lncRNA with the capability to control the chemoresistance and metastasis of CRC through regulating the levels of miR‐181a‐5p expression and the activation of the Wnt/β‐catenin signalling pathway.^24^ Another lncRNA H19, which is overexpressed in patients of CRC, can regulate the expression levels of β‐catenin by competing with miR‐200a, thus resulting in more activated proliferation and metastasis of cancer cells.^25^ In addition, as a ceRNA of miR‐21, lncRNA GAS5 can regulate the levels of miR‐21 expression in cells of hepatocellular carcinoma.^26^ CASC7 is an lncRNA enriched in nuclear fractions.^10^, ^27^ In SH5Y‐SY cells pretreated with OGD/R, CASC7 promoted the pre‐processing of NaSH, while the knockdown of CASC7 expression by siRNA can up‐regulate the expression of miR‐30c, thus promoting the apoptosis of cancer cells. As a family member of miR‐30s, miR‐30c plays an essential role to induce the onset and progression of various types of tumours, including colon cancer, liver cancer and breast cancer.^28^, ^29^, ^30^, ^31^ As a tumour suppressor, miR‐30c can also inhibit the migration, proliferation and metastasis of cancer cells.^28^, ^32^, ^33^, ^34^ In this study, we enrolled HF patients with preserved ejection fraction and reduced ejection fraction. Then, we performed qPCR to analyse the expression of several miRNAs and lncRNAs in the plasma and peripheral blood monocyte samples collected from these patients. We found that miR‐30c expression was inhibited in HFpEF and HFrEF groups, while the lncRNA‐CASC7 expression was enhanced in HFpEF and HFrEF groups. In addition, we performed an ROC curve analysis to evaluate the potential values of above miRNAs and lncRNAs in the diagnosis of HF. MiR‐30c had an AUC of 0.62 while lncRNA‐CASC7 had an AUC of 0.85, both showing capability great value in differentiating HF patients from controls.

Interestingly, miRNAs can regulate the expression of genes responsible for heart fibrosis. For example, both miR‐30 and miR‐133 are down‐regulated during HF. Since miR‐30 and miR‐133 can suppress the expression of CTGF, the reduced expression of miR‐30 and miR‐133 promotes the fibrosis of heart tissues in HF.^12^

Several miRNAs can regulate the expression of Pak1 and Cdc42. For example, The suppression of miR‐30c expression in cardiomyocytes increased the expression of Pak1 and Cdc42, indicating that miR‐30c is an important regulator of Pak1 and Cdc42 expression.^35^ Also, in the pathogenesis of Ewing Sarcoma, a highly aggressive bone tumour, miR‐130b was reported to induce proliferation, invasion, and migration in vitro and increase metastatic potential via activation of the Cdc42 and Pak1 pathway.^36^

Abonnenc et al^37^ showed that cardiac fibroblasts have high expression of miR‐30c. In addition, serum level of miR‐30c expression can be used as a novel marker to estimate the cardiotoxicity of bevacizumab. During the progression of lung cancer, the level of miR‐30c expression remains stable over time.^34^ Therefore, the level of miR‐30c expression in serum can be used to estimate the cardiotoxicity of different drugs. Both miR‐133 and miR‐30 can negatively regulate the progression of fibrosis by directly interacting with CTGF, which is a critical regulator in the synthesis of proteins associated with fibrosis.^38^ In HF patients, the fibroblasts and cardiomyocytes in their heart tend to secrete a high amount of CTGF, which in turn promote the progression of HF. Since miR‐133b and miR‐30c can regulate the expression of CTGF in both fibroblasts and cardiomyocytes, they exert a protective effect against the progression of HF.^39^ In this study, we carried out a luciferase assay to explore the inhibitory effect of miR‐30c on lncRNA‐CASC7, lncRNA‐CCAT1 and lncRNA‐AK017368 expression. MiR‐30c significantly repressed the luciferase activity of wild‐type lncRNA‐CASC7 but showed no obvious inhibitory effect on the luciferase activity of lncRNA‐CCAT1 and lncRNA‐AK017368. Furthermore, we overexpressed lncRNA‐CASC7, lncRNA‐CCAT1 and lncRNA‐AK017368 in H9C2 cells and checked the expression of miR‐30c. The transfection of lncRNA‐CASC7 remarkably repressed the expression of miR‐30c.

IL‐11 is implicated in hematopoiesis^40^ and tumorigenesis.^41^ To be specific, hematopoietic stem and progenitor cells were reported to respond to different functional demands by the activation of IL‐11 pathway. Also, IL‐11 has a stronger correlation with the progression of sporadic and inflammation‐associated colon and gastric cancers. In fact, IL‐11 plays a strong pro‐fibrotic role in cardiac fibroblasts to increase the synthesis of ECM by cardiac fibroblasts, thus affecting the migration and invasion of cardiac fibroblasts.^42^, ^43^

However, there are limitations of our study. On one hand, the samples used in our study were the patient plasma samples and PBMCs, while it would be more convincible if myocardial tissues could be collected and used in this study. On the other hand, the sample size was limited, which will influence the accuracy of our study. Therefore, in our future study, larger sample size should be applied with more appropriate patient tissue, that is myocardial tissue, should be collected for investigation.

## 5 CONCLUSION

In summary, the expression of CASC7 was high while the expression of miR‐30c was low in plasma and peripheral blood monocytes collected from HF patients. Furthermore, miR‐30c can be used as a diagnostic biomarker of HF, while CASC7 is a competing endogenous RNA of miR‐30c. CASC7 can indirectly regulate IL‐11 expression via miR‐30. The results of this study provided a new perspective for lncRNA‐directed diagnosis and treatment of HF.

## CONFLICT OF INTEREST

The authors declare that they have no conflict of interest.

## AUTHOR CONTRIBUTION

**Yu‐li Xu:** Conceptualization (equal); Formal analysis (equal); Project administration (equal); Writing‐original draft (equal). **Yang Liu:** Conceptualization (equal); Software (equal); Supervision (equal); Writing‐original draft (equal). **Ru‐ping Cai:** Investigation (equal); Methodology (equal); Supervision (equal). **Shi‐rong He:** Investigation (equal); Software (equal); Visualization (equal). **Ri‐xin Dai:** Investigation (equal); Visualization (equal). **Xi‐heng Yang:** Investigation (equal); Software (equal). **Bing‐hui Kong:** Investigation (equal); Methodology (equal). **Zhen‐bai Qin:** Resources (equal); Software (equal); Visualization (equal). **Qiang Su:** Conceptualization (equal); Funding acquisition (equal); Project administration (equal); Supervision (equal); Writing‐review & editing (equal).

## CONSENT FOR PUBLICATION

Not applicable.

## ETHICS APPROVAL AND CONSENT TO PARTICIPATE

This study was approved by the Ethics Committee of Affiliated Hospital of Guilin Medical University and informed consent has been obtained upon the initiation of this study.

## Data Availability

The data that support the findings of this study are available from the corresponding author upon reasonable request.
