#  

**DOI:** 10.1111/jcmm.17199

**Published:** 2022-04-05

In Xu et al.,^1^ the published article contains an error in Figure 9F. The correct figure is shown below. The authors confirm all results and conclusions of this article remain unchanged.

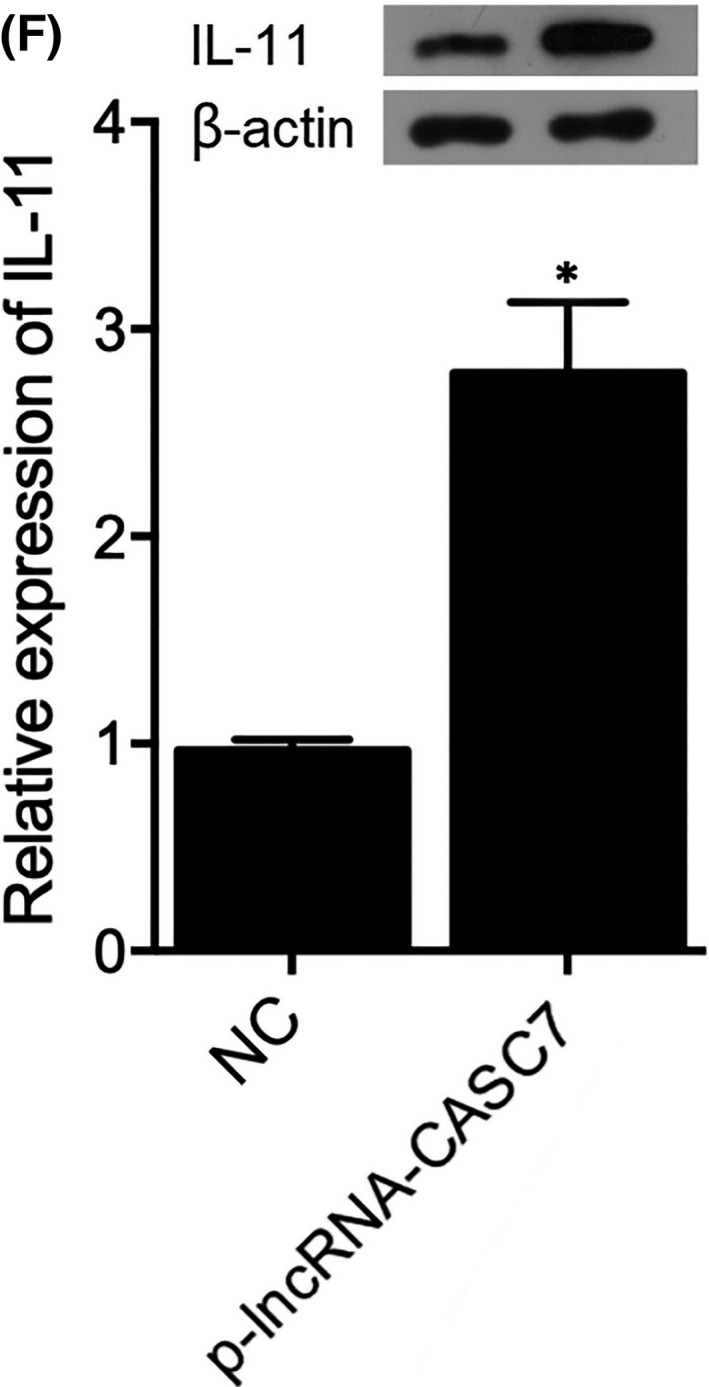

FIGURE 9: lncRNA‐CASC7 suppressed the expression of miR‐30c in H9C2 cells (*p value < 0.05 vs. NC group; NC: negative control). F, Overexpression of lncRNA‐CASC7 increased the expression of IL‐11 in H9C2 cells

## Supporting information

Supplementary Material
