# Supplementary material
Source: J Cell Mol Med. 2022 Apr 5;26(7):2133. doi: 10.1111/jcmm.17199 (PMC8980894; doi:10.1111/jcmm.17199)

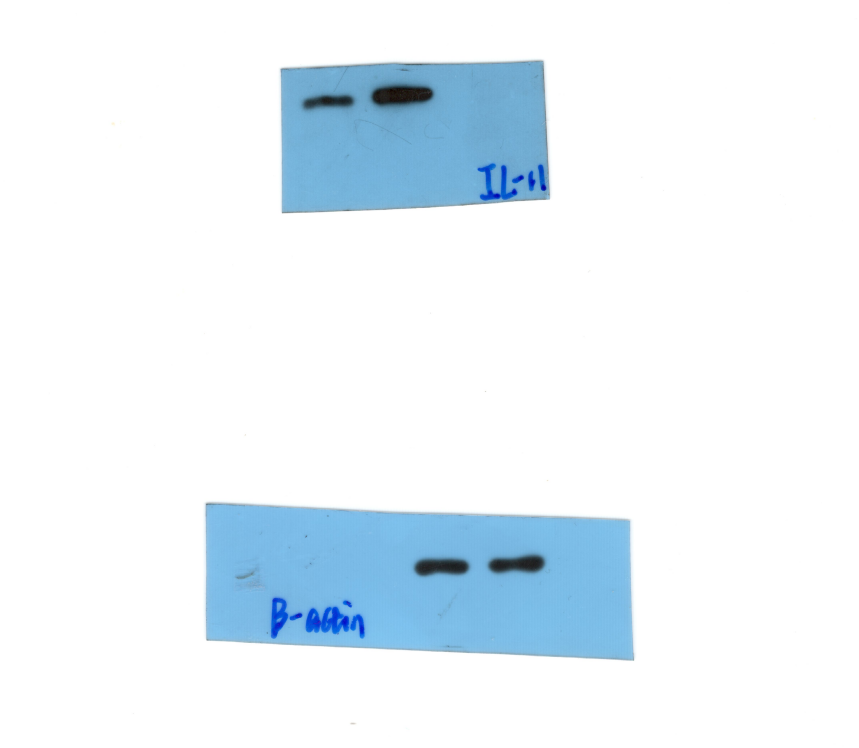

Supplement: Supplementary file 1 — Supplementary Material [file JCMM-26-2133-s001.png]
